# Neighborhood Influences on Women’s Parenting Practices for Adolescents’ Outdoor Play: A Qualitative Study

**DOI:** 10.3390/ijerph16203853

**Published:** 2019-10-12

**Authors:** Maura M. Kepper, Amanda E. Staiano, Peter T. Katzmarzyk, Rodrigo S. Reis, Amy A. Eyler, Derek M. Griffith, Michelle L. Kendall, Basant ElBanna, Kara D. Denstel, Stephanie T. Broyles

**Affiliations:** 1 Prevention Research Center, Washington University in St. Louis, 1 Brookings Drive, St. Louis, MO 63130, USA; reis.rodrigo@wustl.edu (R.S.R.); aeyler@wustl.edu (A.A.E.); basant.elbanna@wustl.edu (B.E.); 2 Pennington Biomedical Research Center, 6400 Perkins Road, Baton Rouge, LA 70808, USA; Amanda.staiano@pbrc.edu (A.E.S.); peter.katzmarzyk@pbrc.edu (P.T.K.); kara.denstel@pbrc.edu (K.D.D.); stephanie.broyles@pbrc.edu (S.T.B.); 3 Center for Medicine, Health and Society, Vanderbilt University, 2301 Vanderbilt Place, Nashville, TN 37235, USA; derek.griffith@vanderbilt.edu; 4 School of Public Health, Louisiana State University Health Sciences Center, 2020 Gravier Street, New Orleans, LA 70112, USA; mkenda@lsuhsc.edu

**Keywords:** neighborhood, parenting, physical activity, outdoor play, adolescent, adolescent wellbeing

## Abstract

Understanding factors that influence parenting decisions for outdoor play is necessary to promote physical activity during critical years for adolescent adjustment. This study explored physical and social environmental influences on parenting decisions and rules for their child’s outdoor play using semistructured in-depth interviews with parents (*n* = 30, 29 of whom were mothers) of adolescents. Mothers from low- (*n* = 16) and high-disadvantage (*n* = 13) neighborhood environments were recruited to identify environmental factors that resulted in parenting decisions that either promoted or hindered outdoor play and identify differences across neighborhood types. Data were analyzed using a grounded theory approach. Mothers limit their child’s independent play, as well as the location and time of outdoor play, due to both social and physical aspects of their neighborhood. Seven themes (safety, social norms, sense of control, social cohesion and neighborhood composition, walkability, and access to safe places for activity) were identified as influencers of parenting practices. Mothers in high-disadvantage neighborhoods reported facing greater neighborhood barriers to letting their child play outside without supervision. Physical and social neighborhood factors interact and differ in low- and high-disadvantage neighborhoods to influence parenting practices for adolescent’s outdoor play. Community-level interventions should target both physical and social environmental factors and be tailored to the neighborhood and target population, in order to attenuate parental constraints on safe outdoor play and ultimately increase physical activity and facilitate adolescent adjustment among developing youth.

## 1. Introduction

Physical inactivity is a major public health concern, as it is contributing to a rise in obesity and related conditions, such as diabetes in children [1,2]. The decline in outdoor play, particularly unsupervised or independent play, among today’s children and adolescents contributes to physical inactivity [3,4,5]. Low levels of outdoor play have implications for children’s development beyond physical inactivity, as it enables cognitive, physical, emotional, social and motor learning, which are factors of adolescent adjustment [6,7,8,9,10]. However, physical and social aspects of the neighborhood environment may be limiting a child’s opportunity for outdoor play.

Residence in lower-income, disadvantaged neighborhoods has been associated with lower levels of physical activity and adolescent adjustment, and these neighborhoods are disproportionately inhabited by African American families [11,12,13,14,15,16]. These relationships are explained in ecological models of behavior, which posit that macrolevel environmental factors (e.g., neighborhood disadvantage) may have a direct effect on physical activity behaviors, but this effect may be modified by microlevel environmental factors, such as access to high-quality resources for physical activity [17,18]. Disadvantaged neighborhood environments are more likely to lack access to quality greenspace, parks or playgrounds and well-maintained sidewalks and have incivilities such as litter, graffiti, and dilapidated homes that affect physical activity [19]. Subsequently, a poor physical environment may influence outdoor play through its impact on the neighborhood social environment, such as the ability of neighbors to form social relationships and build trust. Social environmental factors are less commonly studied in relation to youth’s physical activity; nonetheless, there is evidence that children who live in neighborhoods where parents perceive there are higher levels of social cohesion acquire more physical activity [20,21]. The intersection between the physical and social environment and mechanisms (e.g., parenting practices) by which aspects of the neighborhood environment interact to influence adolescent’s outdoor play is not clear yet is particularly important among adolescents who are more likely to be influenced by their environment, directly or indirectly through parents, and are in critical years of development [19].

Parents are key players in their adolescents’ adjustment and health-related behaviors, including outdoor play and overall physical activity. Parenting practices and styles are related to adolescent adjustment [22,23]. Similarly, parents may support physical activity through encouragement, modeling or sharing in physical activity, or instrumental support (i.e., money for registration fees, transportation to activities, or equipment), but they may also limit their child’s ability to be physically active [24,25]. One way parents may limit physical activity is by restricting outdoor play with rules regarding where their child can play outdoors (e.g., restriction to the backyard only), at what time of day they are able to play outside (e.g., during daylight hours only), or who their child can play with or be supervised by. Children who are free to play outdoors and travel actively without adult supervision accumulate more physical activity than those who are not, yet the neighborhood environment may make parents uncomfortable with unsupervised play [3,4,5]. Parents were more likely to restrict or eliminate opportunities for unsupervised outdoor play if they perceived their child was at risk of being harmed, and these adolescents had lower levels of active transport and moderate-to-vigorous physical activity (MVPA) outside of school hours [26]. However, it is not clear what factors in the neighborhood environment are moderating the relationship between parental behaviors and adolescent outdoor play and can be modified to make parents feel their adolescents are safe to play outside either with friends or siblings or independently. Therefore, this study explored what factors of the neighborhood physical and social environment are perceived as important for parenting practices among parents from neighborhoods characterized as low- and high-disadvantage using an index comprising markers of poverty (e.g., % of households below the poverty level, % receiving public assistance, % with less than a high school degree, % African American, etc.) from United States census data.

## 2. Materials and Methods

### 2.1 Participants

Parents (*n* = 30) of adolescents who were participating in the Translational Investigation of Growth and Everyday Routines in Kids (TIGER Kids) Study (USDA 3092-51000-056-04A) at Pennington Biomedical Research Center (PBRC) in Baton Rouge, Louisiana, were recruited to participate in semistructured in-depth interviews. Adolescents were eligible for the TIGER Kids study if they were between ages 10 and 16 years at their baseline visit, weighed <500 pounds, were not pregnant, were not on a restrictive diet due to illness, and did not have significant physical or mental disability. Parent interviews occurred between 1 month and 13 months after the TIGER Kids baseline visit; therefore, adolescents ranged in age from 10 to 18 years at the time of the qualitative interview.

Parents were purposively recruited by neighborhood type (low- and high-disadvantage) and race (African American and White) to gain diverse perspectives and identify neighborhood factors that positively and negatively influence parenting decisions for outdoor play. The neighborhood environment of eligible participants was characterized a priori by geocoding each participant’s home address using geographic information systems (GIS). Home address was collected via study questionnaire, and these data were managed in REDCap electronic data capture hosted at PBRC [27]. The census block group around each participant’s home address was characterized as low- or high-disadvantage using the concentrated disadvantage index (CDI), which includes percentage of individuals below the poverty line, percentage of households receiving public assistance, percentage female-headed households, percentage unemployed, percentage less than age 18, and percentage African-American [28,29]. A z score was used to calculate low and high CDI for the TIGER Kids study sample using a median split (*n* = 342).

### 2.2. Data Collection

Semistructured in-depth interviews were conducted with parents (e.g., mothers) in a private room in a clinic at PBRC by trained qualitative researchers over a three-month period (April–June 2018). Written consent was obtained prior to each interview. Each participant received $25 for their time and contributions to the study. Ethical approval for this study was provided by the PBRC Institutional Review Board (Washington University in St. Louis IRB #201807166Pennington Biomedical Research Center IRB# 2017-017), and parents provided their written consent to participate. Interviews were focused on parenting decisions/rules around outdoor play for their child who participated in the TIGER Kids study, as well as how and what aspects of their neighborhood environment influence these parenting decisions/rules. The environment was defined using Sallis’ definition of ecology; therefore, questions were asked about people’s transactions with their physical and sociocultural environments [30]. Questions were asked about the child who participated in the TIGER Kids study only. Open-ended questions were generated and refined with the collaboration of the research team and by piloting the interview guide among two employees of PBRC who had a child between the ages of 10 and 18 years. Amendments were made to the interview guide throughout the course of the interviews to gain a clearer understanding of key concepts. Broad, open-ended questions were posed, followed by more specific probing questions to gain detailed data about topics brought up by the participants. The interviewers used reflexive practice journaling of thoughts, impressions, and potential biases after each interview. These thoughts were discussed between the two interviewers on a weekly basis in an effort to reduce potential biases.

### 2.3. Data Analysis

All interviews were audio-recorded and professionally-transcribed verbatim. Interviews were anonymized prior to analysis using NVIVO v11 (QSR International, Melbourne, Australia) and methods consistent with qualitative research standards. Data analysis was guided by principles of thematic analysis that recognizes the importance of using both inductive and deductive strategies [31,32]. Thematic analysis was chosen because the goal of the study was to identify which neighborhood factors influence a parent’s decisions/rules for their child’s outdoor play in the neighborhood.

To help to ensure the integrity of the data analysis, guidelines by Hennink and colleagues (2011) were followed when collecting and analyzing the data [33]. First, four diverse interviews (high and low disadvantage, African American and White, male and female child) were chosen for two trained staff members to memo, discuss, and establish an initial codebook using open coding techniques [34]. In addition to inductive codes, previous research on neighborhood environmental influences on physical activity and topics from the interview guide were used to create deductive codes. Next, focused coding [34] and the constant comparison method [35] were used to generate inductive codes. All coding discrepancies were discussed until an initial codebook detailing code definitions was agreed upon. An additional interview was coded by two staff members to refine the codebook, and inter-rater reliability was calculated. This process was repeated until saturation was met (after 8 interviews). At this point, a final codebook and protocol were established, and all remaining interviews were coded. Two raters independently coded six of these interviews to calculate inter-rater reliability. Across all codes in these six interviews (*n* = 25 codes), raters had an agreement of 95.0%. Once all interviews were coded, codes were grouped into social and physical environmental categories. Additionally, data were compared across the deductive subgroups of low- and high-disadvantage neighborhood that were established in the study design and recruitment (Hennink, Hutter, & Bailey, 2011) [33]. Examining themes within and across the groups allowed patterns and distinctions to emerge, providing a foundation for explaining differences for those in low- versus high-disadvantage neighborhoods. This comparison showed the strength of the themes among mothers from high- or low-disadvantage neighborhoods and whether themes were expressed differently by mothers in the two groups.

## 3. Results

Interviews took an average of 40 min, ranging from 19 to 61 min. Almost all of the participants (*n* = 29, 96.7%) were mothers, but one participant was a grandmother. Therefore, parents are referred to as mothers in the following results. Half of the mothers were African American (*n* = 15, 50.0%), and fourteen (46.7%) lived in high-disadvantage neighborhoods. Of those who lived in high-disadvantage neighborhoods, 11 (68.8%) were African American. Five mothers (16.7%) reported an annual household income of less than $30,000. Mothers were on average 45 years of age (range = 36–71), and their adolescents were on average 13 years old (standard deviation 2 years). Approximately half of the adolescents were female (*n* = 17, 56.7%) and African American (*n* = 15, 50.0%).

### 3.1. Parenting Practices for Adolescent’s Outdoor Play

Major themes for parenting practices for outdoor play were supervision, time of day, and location of play. Mothers in low-disadvantage neighborhoods were more comfortable with letting their kids play outside without continuous supervision. Most of the mothers in low-disadvantage neighborhoods mentioned that they allow their kids to play in the yard or travel in the block around their house if they or another adult they trust is able to check in intermittently. Conversely, adolescents in high-disadvantage neighborhoods were commonly limited to playing in their own yard, the sidewalk in front of their house, or not allowed to play outside at all. Adolescents who lived in low-disadvantage neighborhoods were more likely to have freedom to play independently throughout the entire neighborhood and were also more likely to have access to community areas (e.g., community pools or basketball courts) where mothers felt comfortable with them playing with siblings or peers. Having other adolescents in the neighborhood or siblings, especially those who were older, helped mothers to relax their rules for outdoor play; this was particularly true when mothers trusted the other parents in the neighborhood. In the low-disadvantage neighborhoods, the mothers were more confident that the adults in the neighborhood look out for children/adolescents playing outside and would be willing to intervene in cases of emergency, in comparison to the high-disadvantage neighborhoods. For adolescents with freedom to play independently, it was required that they ask for permission before they go anywhere and keep the parent informed about where they are at all times. In general, mothers seemed to give a child with a cellular telephone more freedom to move around the neighborhood independently. Each mother reported that her child was only allowed to play outside after dark if they had adult supervision. Additionally, some mothers limited the time of day adolescents play outside to times when traffic volume is lower.

### 3.2. Neighborhood Influences on Parenting Practices for Outdoor Play

**Figure 1:**
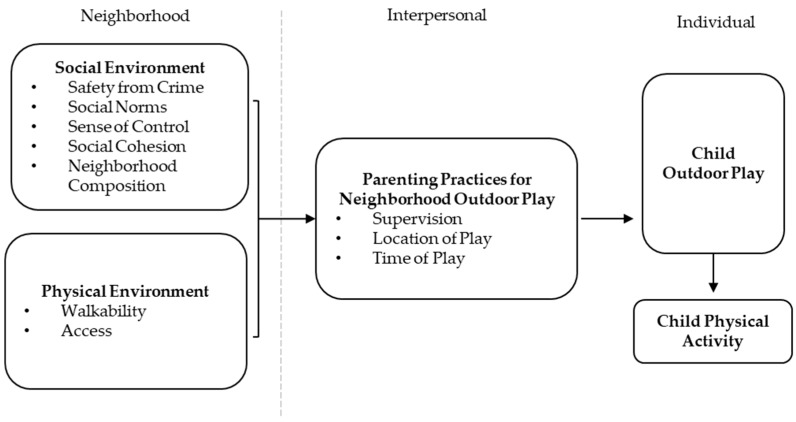
Multilevel influences on children’s outdoor play and physical activity.

Seven themes were identified as influencers of mothers’ parenting decisions and rules for their child’s outdoor play in their neighborhood (Figure 1). Themes were grouped into social and physical environmental factors, and key differences between mothers from low- and high-disadvantage neighborhoods are discussed throughout and presented in Table 1. Social environmental factors that influenced parenting decisions and rules for outdoor play are safety from crime, social norms, sense of control, social cohesion, and neighborhood composition. Physical environmental influencers included walkability and access to safe places for activity.

### 3.3. Social Environment

Theme 1: Safety from Crime

Safety from crime in the neighborhood environment was a highly discussed determinant of mothers’ parenting practices for outdoor play. In general, mothers who did not feel their child was safe from crime in the neighborhood did not let their adolescents play outside without adult supervision. The factors that contributed to mothers’ feelings of safety differed between low- and high-disadvantage environments, and mothers in high-disadvantage neighborhoods commonly felt their child was not safe. Strangers in the neighborhood were a major source of fear for mothers, and mothers from high-disadvantage neighborhoods or neighborhoods that had many access points or through streets were more likely to report them as a threat. One mother felt that the presence of more kids that she did not know being out in the neighborhood during summer months created an unsafe environment for her child. Vacant lots or abandoned homes also made mothers in high-disadvantage neighborhoods feel less safe due to the possibility of squatters. In general, mothers in high-disadvantage neighborhoods reported more serious crimes (i.e., gun violence, theft) as threats to their child’s safety. Factors that mothers felt would make them feel safer in their neighborhood were higher police presence in the neighborhood, formal or informal neighborhood watch, knowing and trusting people in the neighborhood, and reduced access into the neighborhood (i.e., gated community or fewer entrances).

“Every once in a while, we’ve had vagrants or panhandlers in the neighborhood that we’ll see. It’s just a little disconcerting not that there’s anything wrong with being poor or anything, but I don’t know these people.”

—*low disadvantage, white mother.*

“Well my neighborhood seems to be like a safe place and everybody be looking out for each other and that’s why I think. Because three doors down, we got a police that stays there and my neighbor directly across the street from me works for (prison name). He’s a guard over there. And I just think everybody knows each other.”

—*high disadvantage, African American mother.*

“Because we’re in a rural area they… you very seldom see a cop car… because they only have like one or two that go all… and it’s a big parish. And I think they only have two units that patrol regularly.”

—*low disadvantage, white mother.*

Theme 2: Social Norms

Mothers who saw other children/adolescents playing outside in the neighborhood felt more comfortable letting their kids outside to play. In other words, if playing outside was a social norm, mothers were more likely to let their play outside. However, it was important that other children/adolescents were abiding by similar rules for outdoor play as the mother enforces for her child. Mothers reported that having similar principles for outdoor play and for child-rearing in general as other parents in their neighborhood made them more comfortable letting their child play outside. Sharing the same religion was a way that mothers felt more confident that other parents in their neighborhood shared similar values that would translate to parenting behaviors. Within low-disadvantage neighborhoods, mothers generally felt they shared the same values and beliefs and had similar rules around outdoor play as other parents in their neighborhood. By contrast, mothers within high-disadvantage neighborhoods felt they were stricter with their child. Mothers who saw other children/adolescents out in the neighborhood at locations or times that they would not allow and felt that kids roamed freely and had a lack of respect for adults in general were more likely to limit their child’s independent outdoor play because they felt it was important for their kids to be around other kids who have similar values and rules.

“Well, the fact that we have good neighbors makes it easier to let them go outside and play. I see across the street in particular there’s a house with some young kids, and they’re out there frequently playing. That makes me feel better about letting the kids go out and play on their own.”

—*low disadvantage, white mother.*

“I guess it does make me more comfortable because I know that we all, we supposed to be believing in one God, and I hear the way they talk, and so I know our beliefs are pretty much the same. It does make you more comfortable.”

—*low disadvantage, African American mother.*

“You don’t want your kid around anybody. You know, you want them around someone who has the same views as you. Whether they look like you or not, you just want somebody who’s kind and considerate, who are instilling good things in your children. That’s what I want, anyway.”

—*high disadvantage, African American mother.*

Additionally, mothers who felt the neighborhood social norm was to be ‘family-oriented’ were more comfortable with their child playing outdoors; family-oriented was described by the mothers as having adults in the neighborhood who look out for each other’s children, are cognizant of kids playing in the neighborhood when driving and would help their child if they got hurt. Mothers in low-disadvantage neighborhoods reported the norm to be this ‘family-oriented’ approach, unlike mothers in high-disadvantage neighborhoods. Social norms also played a role in rules mothers set for their child by the inherent desire not to be different from other parents in their neighborhood. For example, one mother said:

“I’m not gonna be the only one lettin’ my kids walk around by herself. It keeps you in check. You don’t wanna be the strictest parent, but you don’t wanna be the most lax parent, so, oh yeah. It’s definitely a social norm for you to say, “Okay, well I’m not as bad”. I definitely think that how other people treat their kids, especially in my neighborhood, dictated how we were gonna be. It’s just gave us the limits of what we wanted to be like.”

—*low disadvantage, white mother.*

Theme 3: Sense of Control

Across neighborhood types, mothers who felt they had a sense of control over things occurring in their neighborhood were more comfortable with their child playing outdoors. The majority of mothers felt they had control over crime in their neighborhoods due to neighbors who look out for negative activity. The mothers who felt that they lacked control lived in high-disadvantage neighborhoods or neighborhoods that had strangers passing through regularly. One parent who lived in a high-disadvantage neighborhood stated:

“I don’t feel like I have that much control and that I’m that close with my neighbors, so I just stay strict the way I am.”

—*high disadvantage, African American mother.*

Factors that helped individuals to feel they had a sense of control were familiarity and communication with neighbors, knowing that their neighbors were keeping an eye out, fewer rental homes, police officers that live in their neighborhood, and few entrances/exits to the neighborhood. If individuals were able to identify or keep out strangers, they felt more in control. Across neighborhood types, web applications, e.g., Facebook neighborhood groups and Nextdoor, were effective ways to communicate with neighbors that increased control over negative things occurring in the neighborhood, such as petty thefts, robbery, traffic, dog waste/litter, etc. Mothers who live in low-disadvantage neighborhoods mentioned that a neighborhood association was a means of control.

“So it’s like they keepin’ an eye out for everybody, and we know who belongs on the street and who doesn’t. I guess that’s the plus for livin’ on a dead end. So you see the same cars and the same people often.”

—*high disadvantage, African American mother.*

Theme 4: Social Cohesion

Almost all of the mothers reported that how well they know their neighbors and their level of trust with neighbors influences their parenting practices for their child’s outdoor play. Only when mothers trusted their neighbors were they willing to let other adults in the neighborhood supervise their child playing outside or allow them to play in their neighbor’s yard. However, mothers typically described their interaction with neighbors as superficial (e.g., saying hello when they passed). There was little difference in the extent of neighbor relations between high- and low-disadvantage neighborhood.

“People that we knew really, yeah. It might have been maybe I would say four to five people’s homes that we felt comfortable enough for them to just go on a Saturday afternoon or whatever if we had time.”

—*low disadvantage, white mother.*

When asked what has helped or would help them to establish relationships with their neighbors, mothers reported the following: number of years living in neighborhood, attending the same church, participating in neighborhood events and association meetings, watching sporting events together, and having a dog/being outside in the neighborhood and having a communal space (e.g., community pool or park) where neighbors could gather. Although they facilitated communication, web applications (e.g., Facebook and Nextdoor) did not make mothers feel they knew or trusted their neighbors. Barriers to getting to know neighbors were lack of time at home due to employment or kids’ activities outside of school, introverted personality and feelings that they would not fit in with other parents in the neighborhood. Overall facilitators of neighbor relations were more common in low-disadvantage neighborhoods, particularly access to communal spaces and participation in community events and associations. Mothers across neighborhoods expressed interest in getting to know other parents in their neighborhood and wanted more events to happen in the community.

“More get-togethers. We have lived there 18 years. We’ve never had a party or—they have these progressive dinners at some of these neighborhoods and gettin’ to know your neighbors better so that you would feel comfortable with your kids going there.”

—*low disadvantage, white mother.*

“If you have more adults involved, we could sit outside and that would allow the kids more time to play because that way you can watch them, what they’re doing. You don’t have to worry about where your kids are with or if they’re with strangers.”

—*high disadvantage, African American mother.*

Theme 5: Neighborhood Composition

Two themes within neighborhood composition were important for parenting practices for outdoor play: the presence of other children/adolescents and having older neighbors. As mentioned in the social norms section, seeing other children/adolescents playing outdoors generated a level of comfort for mothers to also allow their child to play outside. However, mothers mentioned that there were not many peers in their neighborhood that were the same age as their child, which ultimately decreased their child’s desire to play outside. This was particularly true for families in rural areas where neighbors were limited and far apart. On the other hand, mothers mentioned that their neighbors were mostly of older age. Having older neighbors was desirable to many mothers because they felt they looked out for the children/adolescents in the neighborhood and were at home more (e.g., when children are returning home from school). These ‘eyes on the street’ made them feel safer with their child being outside (particularly walking home from the bus stop independently).

“Most of the people that live across the street from the park are older people and they sit. They have a screened-in porch, so they’re always sittin’ outside. So if somethin’ was to happen, at least you have an adult, if they’re out there at that time that could see what happened.”

—*high disadvantage, African American mother.*

### 3.4. Physical Environment

Theme 6: Walkability

All mothers who reported living on a street with a high traffic volume indicated that this greatly interfered with letting their child play outside. These mothers were more likely to limit play to the backyard, if available. Mothers in high-disadvantage neighborhoods commonly reported that they lived on a busy street or a street that was close to the highway or interstate. Although mothers living in low-disadvantage neighborhoods mostly lived on ‘quiet’ streets, they still expressed interest in changes to the traffic in their neighborhood that would make it safer for their child to play outside. For example, mothers wanted to close streets for play during designated times, make some streets one way, gate their community to reduce through traffic, decrease speed limits, or encourage neighbors to put signs in their front yards when children are outside playing. Mothers who reported living in rural areas (*n* = 3) did not allow their child to leave their property because they lived off of rural roads with high speed limits and no sidewalks.

“She has to stay in the yard, because we are on a busy street. Don’t get too close to the road, that kind of thing.”

—*low disadvantage, white mother.*

“The neighbors are really nice but again no sidewalks to walk on or anything and some of the younger people drive like maniacs through there.”

—*high disadvantage, white mother.*

“I guess find a way to enforce the speed limit or close some streets so that there’s places where kids can play and places where you can drive and just don’t do both on the… in the same place.”

—*low disadvantage, white mother.*

Other factors that were important for neighborhood walkability across low- and high-disadvantage neighborhoods included the presence of well-maintained sidewalks that connect throughout the neighborhood; bike lanes or a shoulder on the roads; walking trails; attractions within walking distance (e.g., community pool, parks, businesses); and dogs being contained. The connectivity of neighborhood was cited as both positive and negative. If the streets did not connect (e.g., many dead ends), it forced adolescents to get on a busy or main street while riding their bike around the neighborhood or walking to a friend’s house to play. On the other hand, mothers liked less connectivity because it reduced through traffic and presence of strangers in the neighborhood.

“If it was a busy street or cut through, they would’ve never been allowed to play in the front yard without us bein’ right there alongside of ‘em. I probably would’ve made ‘em stay in the back yard, so I definitely feel the structure of our neighborhood and the fact that we do have sidewalks definitely impacted them having more freedom than if they hadn’t.”

—*low disadvantage, white mother.*

“I mean it has sidewalk. Where we’re located it’s a little harder to make a path, because some of the streets are dead ends, so to kind of make a walking path you have to go on up a street that’s a lot more busier.”

—*low disadvantage, white mother.*

Theme 7: Access

Across both neighborhood types, mothers expressed the desire to have access to resources like a safe park, open greenspace (e.g., schoolyard that is open to the public), a community pool, and basketball court within walking distance, and mothers felt that having this access would encourage them to let their kids play outside more frequently. Mothers who lived in subdivisions or new housing developments, all of which were low-disadvantage neighborhoods, reported having sidewalks, trails, and access to play equipment and communal areas (e.g., community pool, basketball courts and clubhouse) that promoted outdoor play. However, among mothers who had parks, safety within the park and/or the need to cross a railroad or main road to get to the park were barriers to independent play. Mothers in high-disadvantage neighborhoods mentioned that on many occasions, the kids used an empty lot, church yard, or open schoolyard to play. Nevertheless, in most cases, these spaces were either not available or had limitations (e.g., only open during certain hours or mothers felt they should not use them). In addition to high-disadvantage neighborhoods, rural areas, all of which were low-disadvantage, lacked access to communal areas, which mothers felt negatively influenced their child’s outdoor activity.

“I wish they had sidewalks and I wish they had a place, ya know, like a park where you could go to BBQ or just, ya know, go on swings or something like that. It would be nice, ya know, just ya know maybe a basketball court or something like that.”

—*high disadvantage, African American mother.*

“I wish there would’ve been, like you were sayin’, more green spaces for them to go that weren’t private property, like at the church. It always felt like, “Yeah, the church allows it, but I always felt we gotta be super careful ‘cause it’s private property kind of thing. I wish it was a public park.”

—*low disadvantage, white mother.*

“We live in a very rural area. There’s no playgrounds, no basketball courts. There’s really nothing for kids to do in that area. It’s more… it’s mainly like older, retired people that live there.”

—*low disadvantage, African American mother.*

“It’s a new development so there are planned walking trails and sidewalks and a neighborhood pool and a neighborhood activity center, in the center. The neighbors are friendly and it’s designed so that people get out. People are on their porches. People are walking. There’s ponds, lots of little ponds and fountains. So people are walking, strollers and babies and dogs. Everybody has a dog so everybody’s out. It’s sort of designed for, I guess, healthy living in a sense because people are encouraged to be outside.”

—*low disadvantage, white mother.*

## 4. Discussion

This study explored which ecological aspects of the neighborhood environment influence parenting practices and may result in restrictions on adolescent’s outdoor play and reduce overall levels of physical activity. This understanding is important for adolescent adjustment as outdoor play and physical activity has been related to social, emotional, and cognitive wellbeing among adolescents [9,36]. Interviews demonstrated that mothers are limiting independent play and the location and time of outdoor play due to their perceptions of and reactions to social and physical neighborhood factors.

Seven aspects of the social and physical environment were identified as important for parenting practices around outdoor play: safety from crime, social norms, sense of control, social cohesion, neighborhood composition, walkability, and access to safe places for activity. Our findings are consistent with previous research showing direct relationships between the built or physical environment and adolescents’ physical activity [19,37,38]. The physical characteristics of a neighborhood, such as land-use mix and residential density, are important for promoting activity among children and adolescents and were identified by mothers as relevant for parenting practices [19]. Differences in neighborhood design may partially explain key differences in parenting practices reported by mothers in rural versus urban settings. There is less evidence for the direct relationships between the social environment and youth physical activity, yet several components identified as important for parenting practices in these interviews have some quantitative support [39,40]. For example, numerous research studies in moderate to large child/adolescent populations reported positive associations between parental perceived social cohesion and control (i.e., collective efficacy) and physical activity [41,42,43,44]. In particular, adolescents whose mothers perceived higher levels of collective efficacy in their neighborhood played outside for longer periods of time, watched less television, and visited the park or playground more frequently [43]. Despite substantial evidence, social factors are seldom taken into account when promoting outdoor play and are subsequently underutilized in interventions [45]. An important implication from this work is that social and physical environmental themes were not mutually exclusive. Safety, for example, was directly linked to parenting practices while also influenced by several other environmental factors, such as social cohesion, sense of control, traffic, etc. Research has increasingly supported that aspects of the social environment, such as social cohesion and control, are linked to crime and health outcomes, including obesity, self-reported health, and mortality [46]. Interactions such as this have long been supported by ecological models that acknowledge the interaction within and across levels of influence on an individual’s behavior. Understanding the interaction between physical and social environmental factors becomes relevant when identifying factors on which to intervene as the order in which change occurs may be relevant (e.g., access to quality locations for play may be necessary before action to establish social relationships) and positive or negative unintended changes may occur from intervening on a single environmental construct [47].

This research identified that challenges to letting adolescents play outside without supervision differed between mothers from high- and low-disadvantage neighborhoods. Overall, mothers in high-disadvantage neighborhoods faced greater neighborhood barriers, which resulted in adolescents being limited to play in their own yard, the sidewalk in front of their house, or not allowed to play outside at all. By contrast, adolescents in low-disadvantage neighborhoods were able to play in their yard or in the block around their house. This difference was expected as previous research has demonstrated that neighborhoods inhabited by low-income individuals are less supportive for being active (e.g., lack parks and playgrounds, higher crime) [19]. Nonetheless, this is the first study to uncover which environmental factors must change in low- and high-disadvantage neighborhoods for mothers to allow their adolescents to play outside independently. Despite key differences, interviews indicated that adolescents in both low- and high-disadvantage neighborhoods were rarely allowed to play freely throughout their neighborhood or in common areas (e.g., parks, neighborhood streets), suggesting that both high- and low-disadvantage neighborhoods could benefit from tailored approaches. Interventions should be tailored to target neighborhood factors that are necessary and desired by parents within the community in order to achieve equal opportunities for independent outdoor play in all neighborhoods and populations.

This work demonstrates the need for multifaceted and multilevel approaches that cross disciplines, organizations, and cultures to promote outdoor play through change in parenting practices. Environmental and policy-level interventions may be necessary to address mothers’ desire for access to safe places to play, well-maintained sidewalks, traffic calming measures (e.g., speed limit reductions or bumps) or limited neighborhood access. The Task Force on Community Preventive Services has recommended urban design, land use, and transport interventions that address these factors and have been shown to effectively promote physical activity. However, the ability of these interventions to change parenting practices to increase adolescents’ outdoor play, as opposed to physical activity in general, has not been explored [48]. Although effective and wide-reaching, these interventions are often challenging due to the financial requirements and the cross-sector (i.e., communities, transportation officials, community planners, health professionals, and governments) effort required [48]. Community-level, grass-roots interventions may be better suited to address several factors that were also identified as important for parenting practices for outdoor play. For example, mothers expressed desire for increased community events in order to get to know and trust their neighbors (build social cohesion), for the use of signs to indicate children/adolescents are playing to slow traffic, or for community watch programs to keep eyes on the street and increase a sense of control. Furthermore, it has been argued that capitalizing on the inherent social structures in our societies may provide a cost-efficient way of encouraging people to be physically active and better use the built environment in which we live. Open Streets initiatives, like Ciclovía programs or Play Streets, are an example of an existing intervention that made a change to the physical environment (temporary street closures from motorized vehicles) that resulted in a corresponding change to the social environment (increased social cohesion) and individual behavior change (increased physical activity). Furthermore, Open Streets addressed two themes identified as important for parenting practices in these interviews: social cohesion and access. However, routinely held events, which are likely necessary for continued behavior change, may be difficult to sustain [49,50]. Nonetheless, it is promising that multisector approaches have the potential to simultaneously address multiple influences which may interact to alleviate parents’ concern, change parenting practices, and increase independent outdoor play.

Approaches to effectively reach mothers were identified through this research. The use of web-based applications (e.g., Facebook groups or Nextdoor) or neighborhood associations may be effective channels to raise awareness and share information among neighbors that would facilitate increased social cohesion and sense of control and ultimately increase independent outdoor play. “Social” apps that use behavior change techniques, such as providing opportunities for social comparison and social support, to promote physical activity among adolescents are promising but lack robust scientific evidence for effectiveness, reach, and sustainability [51]. Furthermore, apps have not been explored as a mode to change ecological constructs, such as social cohesion and control. This research also indicates that faith-based avenues may increase mothers’ sense of similar values and beliefs among neighbors that would result in mothers’ comfort with unsupervised peer play.

This understudied topic warranted the use of qualitative methods that provided mothers’ perspectives on how and which neighborhood factors are influencing their parenting decisions for outdoor play. However, there are limitations inherent with qualitative research. Response bias may have occurred, particularly because interviewers lacked diversity in terms of race and neighborhood environment. First, we only interviewed mothers in Southeast Louisiana who participated in the TIGER Kids research study; therefore, results may not be generalizable to all states or to fathers. Parents were represented by women (29 of whom were mothers); and therefore, the male (or fatherly) perspective on which neighborhood factors influence one’s parenting decisions is not understood. This limitation is true of the majority of studies examining parental influences on adolescents’ physical activity. Research has focused on the mother-child relationship and, in general, is lacking on the role of fathers [52]. Further research to understand whether similar factors are important for parents living in other states would be beneficial for creating interventions that are scalable across communities. Second, participants mainly lived in dense, urban settings; however, differences were observed in influences on outdoor play among the few rural mothers. Additional research should explore differences between urban and rural settings to identify interventions that are applicable and effective in rural settings to promote outdoor play [53]. Thirdly, the intersection between neighborhood disadvantage and race should be further researched. It was the intent to recruit a diverse sample of African American and White parents across neighborhood types; however, 69% (*n* = 11) of participants from high-disadvantage neighborhoods were African American mothers, whereas 29% (*n* = 4) of mothers from low-disadvantage neighborhoods were African American. This unequal proportion may be due to the inclusion of ‘percentage of households who are African American’ as a factor included in our definition of disadvantage and evidence that African Americans are more likely to reside in significantly poorer neighborhoods than whites [54]. Many factors that were more commonly mentioned by mothers in high-disadvantage neighborhoods (such as fewer rental homes, police officers that live in their neighborhood, and few entrances/exits to the neighborhood) are often proxies for the percentage of African Americans in a neighborhood [55]. Further research is necessary to determine whether parents of different races and ethnicities (e.g., white vs. African American) living in the same neighborhood environment identify different neighborhood physical and social factors as important for their parenting practices. Additionally, the results may only be applicable to outdoor play of adolescents aged 10 to 18 years, and differences among younger versus older adolescents were not explored. In general, adolescents are given more autonomy as they age, and neighborhood factors may become less threatening to mothers as adolescents mature [26,56]. Lastly, our data were limited to information and perceptions from qualitative interviews that asked mothers about neighborhood factors and did not explore other potential influencers on parenting decisions (e.g., adolescents’ perspectives, desires, and needs). For example, an adolescent’s desire to play video games or watch television may influence parental decisions and rules for outdoor play. Furthermore, adolescents’ views on the neighborhood physical and social environment may be important for parenting practices. Exploration of these additional factors would enrich the conclusions of this research. In spite of these limitations, this study provides a unique perspective on a topic for which there is limited research. Additional research is necessary and should build upon understandings gained from this study to further explore the relationship between neighborhoods, parenting practices, and the impact on adolescents’ outdoor play, physical activity, and adolescent adjustment. Triangulation with additional data sources would generate a broader scope of this topic.

## 5. Conclusions

Components of the neighborhood social and physical environment are influencing parental decisions that limit adolescents’ independent play and the location and time of outdoor play. Neighborhood factors interrelate and differ in low- and high-disadvantage neighborhoods to influence parenting practices for outdoor play. Community-level interventions that target both physical and social environmental factors and are tailored to the neighborhood and population may be needed to reduce parental constraints on outdoor play, increase physical activity, and improve the health and wellbeing of developing youth.

## Figures and Tables

**Table 1:** Key differences in perspectives of low- and high-disadvantage mothers.

Themes	Neighborhood Type	
	**Low Disadvantage**	**High Disadvantage**
**Social Environment**		
**Safety from Crime**	Mothers were mainly concerned by strangers.	Mothers were concerned by strangers, vacant lots/abandoned homes, gun violence, and theft.
**Social Norms**	Mothers felt other parents shared values and beliefs and had similar rules around outdoor play. People in their neighborhood are ‘family-oriented,’ cognizant of kids playing in the neighborhood when driving and would help their child if he/she was in danger.	Mothers felt they had more strict rules than other parents in their neighborhood. Mothers did not like that other children lacked supervision.
**Sense of Control**	Mothers did not feel there were factors that needed to be corrected or controlled. These neighborhoods had organized efforts (e.g., neighborhood associations, neighborhood watch or web-application) that were a means for control.	Mothers felt that there was need for control over crime, traffic, and strangers. Control was informal through eyes on the street or police officers that lived in the neighborhood.
**Social Cohesion**	Mothers had relationships with a limited number of neighbors but trusted their neighbors and were open to having relationships. Their neighborhoods hosted organized events for neighbors.	Mothers lacked relationships with their neighbors and felt they didn’t have time to establish relationships. Most of the relationships are based around communication of what is occurring in the neighborhood.
**Neighborhood Composition**	No major differences.	
**Physical Environment**		
**Walkability**	Traffic was not a major concern because they lived in subdivisions with traffic calming measures and had yards for their children to play.	Mothers reported living on busy streets or close to a highway or major intersection and expressed concerns about the lack of traffic calming measures (i.e., stop signs, speed bumps).
**Access**	Overall, mothers wanted more access to play areas. Some mothers reported living in subdivisions or new housing developments that had sidewalks, trails, and access to play equipment/communal areas.	Overall, mothers wanted more access to play areas. Mothers stated that children used an empty lot, church yard, or open schoolyard to play yet expressed that these locations have limitations.
